# Update on Neuromodulation for Treatment-Resistant Depression

**DOI:** 10.12688/f1000research.6633.1

**Published:** 2015-12-02

**Authors:** Bettina Bewernick, Thomas E Schlaepfer

**Affiliations:** 1 Department of Psychiatry and Psychotherapy, University Hospital, Bonn, Germany; 2 Departments of Psychiatry and Mental Health, Johns Hopkins University, Baltimore, USA

**Keywords:** Depression, vagus nerve, seizures

## Abstract

About 30% of patients suffering from a major depressive disorder do not respond sufficiently to established pharmacological, psychotherapeutic, or somatic treatments. Advances in technology and emerging knowledge about the dysfunctional brain circuits underlying depression have led to the development of different neuromodulation techniques. The aim of the present review is to give an update on noninvasive techniques, such as electroconvulsive therapy (ECT), magnetic seizure therapy (MST), transcranial magnetic stimulation (TMS), and invasive techniques requiring brain surgery, such as vagus nerve stimulation (VNS) and deep brain stimulation (DBS). First, the clinical relevance for therapy-resistant depression, including the current level of evidence, are presented.

Neuroethics is concerned with the ethical, legal and social policy implications of neuroscience. A second focus of the review is the application of fundamental ethical principles, such as patient autonomy, patient well-being and justice to neuromodulation therapies. Due to reduced availability and lacking long-term efficacy data, most patients with treatment-resistant depression face a trial-and-error approach to therapeutics. This contravenes the ethical criteria of patient autonomy and justice. In order to raise the level of evidence, financial support of long-term studies, including large samples and randomized control trials, are necessary.

## Introduction

About 30% of patients suffering from a major depressive disorder do not respond sufficiently to established pharmacological, psychotherapeutic, or somatic treatments ^1^. After nonresponse to two adequate treatment steps, a patient is described as having a treatment-resistant depression, which is associated with illness chronicity, a reduced quality of life, and a higher risk for suicide ^2^. The grade of treatment resistance can be evaluated using different models, for example the antidepressant treatment history form ^3^, or the Thase and Rush Model ^4^. A substantial quota of patients with treatment-resistant depression have an anamnesis of multiple pharmacological and psychological treatment attempts and patients, as well as treating psychiatrists, are desperate for alternative approaches. Patients with treatment-resistant depression cannot be cured quickly ^5^ and 20–80% of patients suffering from treatment-resistant depression face a relapse within 5 years, in spite of maintenance therapy ^1, 6– 9^. It is therefore necessary to evaluate long-term effects (more than 5 years of treatment) in order to be able to assess the risk-benefit ratio for new treatment methods.

## Neuromodulation

Advances in technology and emerging knowledge about the dysfunctional brain circuits underlying depression have led to the development of different neuromodulation techniques. All these techniques attempt to change the brain’s neuronal activity in a more or less focal way. For treatment-resistant patients suffering from major depression, neuromodulation techniques offer a therapeutic option.

In this review, noninvasive techniques, such as electroconvulsive therapy (ECT), magnetic seizure therapy (MST), transcranial magnetic stimulation (TMS), and invasive techniques requiring brain surgery, such as vagus nerve stimulation (VNS) and deep brain stimulation (DBS), are described. The clinical relevance for therapy-resistant depression, including the current level of evidence, is discussed ^10^.

## Neuroethics

Neuroethics is concerned with the ethical, legal and social policy implications of neuroscience ^11^. Fundamental ethical principles relevant for neuroethics are patient autonomy (i.e., the patient has a choice of treatment, gets information about different treatment options, as reflected in the informed consent procedure), patient well-being (physicians should prevent and remove harms, and weigh and balance possible benefits of an action against possible risks) and justice (priority should be given to patients who are most seriously impaired and who will benefit most from the intervention, patients should get access to the best treatment). These ethical criteria are analyzed in the context of the current clinical application of neuromodulation treatments in treatment-resistant depression.

## Treatments

### Electroconvulsive therapy

***Method.*** ECT was developed in 1938 and is the oldest and best evaluated neuromodulation therapy for treatment-resistant depression. An electrical current is administered to the brain through the scalp. Seizures are induced under general anesthesia and muscle relaxation. Usually, a series of seizures (9–12) are given over several weeks, generally 2–3 treatments per week.

***Mechanism of action.*** The mechanism of action is not understood, but the induction of a generalized seizure and the postictal suppression ^12– 17^ are important factors contributing to the antidepressant effect.

***Clinical application.*** ECT is highly effective in treatment-resistant depressive disorders, with 50–80% of patients achieving remission ^18, 19^, and is therefore the most effective acute treatment for major depressive disorder ^20, 21^.

***Efficacy.*** ECT has level I evidence for acute efficacy and relapse prevention, and level II for safety and tolerability ^22^ (see Table 1). Transient cognitive side effects, such as postictal confusion and anterograde amnesia, are frequent and more pronounced in bilateral electrode placement as compared to unilateral electrode placement ^23, 24^. Up to 55% of patients report persistent negative cognitive side effects after ECT ^25^.

**Table 1.** Level of Evidence in Brain Stimulation.

Treatment	Invasive	Chronic treatment	Acute efficacy	Long-term efficacy	FDA approval	Safety
ECT		Maintenance treatment optional	Level 1	Level 1	x	Level 2
MST		Maintenance treatment optional	Level 3 70% acute response	Level 3 50% relapse within 6 months	One Phase 2 trial underway, see Clinical trials.gov NCT00973934	Level 3
rTMS			Level 1	Level 3	2008 MDD nonresponse to one medication in the current episode	Level 1
VNS	x	x	Level 2	Level 2	2005 for chronic or recurrent depression after four antidepressant trials	Level 2
DBS	x	x	Level 3	Level 3		Level 3

DBS, deep brain stimulation; ECT, electroconvulsive therapy; MDD, major depressive disorder; MST, magnetic seizure therapy; rTMS, repetitive transcranial magnetic stimulation; TMS, transcranial magnetic stimulation; VNS, vagus nerve stimulation. Note. Level of evidence according to 10: Level 1 requires >2 randomized controlled trials and/or meta-analysis with narrow confidence interval; Level 2 requires >1 randomized controlled trial and/or meta-analysis with wide confidence intervals; Level 3 requires nonrandomized, controlled prospective studies, case series or retrospective studies.

***Ethical aspects.*** Although very well investigated, effective and safe, ECT is still an underused treatment in treatment-resistant depression ^26^ for several reasons. ECT is still stigmatized because of its different use in the past ^27, 28^ and cognitive side effects are often overestimated, in comparison to cognitive impairment due to depression itself. Clinical staff members are still inadequately trained and face prejudices, such as limitations on the use of ECT in elderly patients. This leads to a reduced availability in hospitals. The idea that every patient should have access to the treatment with the best prognosis is reflected in the ethical principle of justice. For ECT, the ethical principle of justice is not adequately met for the above mentioned reasons.

***Current research and outlook.*** Research has focused on maximizing antidepressant efficacy while minimizing cognitive side effects. Thus, administration techniques (unilateral *vs.* bilateral stimulation, ultra-brief pulse-width stimulation), the role of postictal depression, depth of anesthesia ^29, 30^, the separation of effects on cognition and depression ^31– 33^, and the best algorithm for maintenance therapy are current research questions.

ECT is established as a conventional treatment in treatment-resistant depression with few contraindications. ECT therefore often serves as treatment for the comparison group in studies as the “gold standard” for the evaluation of new treatments (e.g., MST).

### Magnetic seizure therapy

***Method.*** In MST, seizures are induced with magnetic pulses. The clinical procedure (general anesthesia, 9–12 sessions) is similar to ECT. The aim of the development of MST was to minimize cognitive side effects through a more focal induction of seizures ^34^.

***Mechanism of action.*** Similar to ECT, the exact mechanism of action is unknown. In MST, only the superficial cortex is exposed during seizure induction, but the seizure generalizes to broader brain regions ^35^. Imaging studies have found evidence for changes in glucose metabolism in brain regions that have frequently been reported as dysfunctional in depression ^36– 38^.

***Clinical application.*** Clinical application is limited to a few study centers worldwide, because a specially modified device is required.

***Efficacy.*** Only data from open-label pilot studies with small sample sizes in a few research sites are actually available. Efficacy seems to be similar to ECT, but possibly with a superior side-effect profile regarding cognition ^38– 43^ (see Table 1).

***Ethical aspects.*** As long as MST is applied in clinical studies with careful patient selection and information about alternative treatment options is available, the ethical principles of patient autonomy and well-being are fulfilled.

Actually, research in MST is completely controlled by a few companies because special devices are required. In spite of attractive results from small samples, research activities have seemed to diminish. The fear of cognitive side effects is one major obstacle to encouraging patients to undergo seizure therapy. Thus, the development and availability of a potential treatment method with a possible superior side-effect profile is delayed. This contravenes the ethical principle of patient well-being and justice.

***Current research and outlook.*** Best stimulation parameters (e.g., finding the optimal stimulus intensity), the relevance of seizure threshold titration, and the development of devices and coils are current research foci. One controlled double-blind trial (n=20) is underway (Lisanby *et al.*, see the Registry on ClinicalTrials.gov NCT00973934 https://clinicaltrials.gov/). Long-term blinded and controlled studies with larger samples, and studies into the evaluation of relapse rates and the role of maintenance MST are needed.

### Transcranial magnetic stimulation

***Method.*** Transcranial magnetic stimulation (TMS) is a noninvasive therapy option which can be applied to outpatients with treatment-resistant depression.

During repetitive TMS, a fast series of brief pulses of strong magnetic stimuli are applied to the brain. Deep repetitive TMS is a modification of repetitive TMS which can reach deeper cortical regions with a special coil ^44^. The H Coil ^45^ is the only coil whose safety and effectiveness has been tested. This coil is able to change cortical excitability at a depth of up to 6 cm ^46^.

***Mechanism of action.*** Repetitive TMS and deep repetitive TMS produce changes in neuronal excitability. The magnetic field generated at the coil passes unimpeded through the scalp and skull. An electrical current is induced in the underlying tissue which modulates neural activity ^47^. Depending on the parameters of stimulation, cortical excitability can be increased or decreased ^48^.

***Clinical application.*** After the identification of the motor threshold, the coil is moved from the motor cortex to the specific target cortical region. In the treatment of major depressive disorder, the target area is usually the left dorsolateral prefrontal cortex ^49– 51^. In contrast to ECT or MST, no general anesthesia is required. Patients and TMS operators should wear earplugs during TMS. Usually, 10–30 treatment sessions of 15–45 minutes are administered daily in an outpatient setting.

***Efficacy.*** There is evidence for repetitive TMS either as a mono- or add-on therapy for the treatment of moderate treatment-resistant depression (evidence level I) ^52^. In 2008, repetitive TMS was approved by the FDA for the treatment of moderate treatment-resistant depression.

Several studies have investigated the efficacy of deep repetitive TMS in patients suffering from treatment-resistant depression ^49– 51, 53– 57^. Deep repetitive TMS seems to be an effective and safe treatment for patients with treatment-resistant depression (see Table 1).

***Side effects.*** Overall, repetitive TMS is seen as safe without enduring side effects: no long-term neurological, cognitive, or cardiovascular side effects are reported ^58– 61^. Transient headache is the most common side effect after repetitive TMS.

Similar to repetitive TMS, deep repetitive TMS is considered a safe treatment. Scalp discomfort, transient headache and dizziness, insomnia, perceiving an odd smell, numbness in the right temporal and right cervical zone, and (in single cases) generalized seizures have been reported ^44^.

There is no long-term evidence for either repetitive TMS or deep repetitive TMS because most studies are limited to 6–12 weeks (see 22 for a comprehensive review of TMS studies).

***Ethical aspects.*** Although, the FDA has approved repetitive TMS (level I evidence for acute efficacy), this treatment is only available in special centers and patients do not have the opportunity to choose this therapy option even though its clinical evidence has been proven. Both repetitive TMS and deep repetitive TMS seem to have lower response rates in treatment-resistant depression as compared to ECT (Lipsman, Sankar *et al.* 2014), but the side-effect profile seems superior. In addition, TMS can be performed in an outpatient setting without anesthesia. Patients should be given the choice between a less effective but also less risky therapy, and a therapy with a higher risk for side effects and higher efficacy. This fits with the ethical criterion of justice and patient autonomy.

***Current research and outlook.*** Current research foci in TMS are the effect of low- and high-frequency stimulation and laterality issues, and optimizing TMS pulse and train parameters, as well as the influence of the characteristics of the TMS pulse itself (with the help of the controllable pulse TMS device). Little is known about combination therapy (e.g., pharmacotherapy, psychotherapy).

### Vagus nerve stimulation

***Method.*** VNS is an invasive brain stimulation method. A small electrical pulse is administered with an implanted neurostimulator to a bipolar electrode, surgically implanted at the left vagus. The pulse generator is implanted under the skin of the left chest. Intermittent electrical currents are sent from the generator to the vagus nerve and *via* the nucleus tractus solitarius to various regions of the brain. Usually, electrical pulses that last about 30 seconds are forwarded about every 5 minutes from the generator to the vagus nerve; other parameters consist of a current intensity of 0.20 to 2.50 mA, a pulse width of 500 ms and a pulse frequency of 20 Hz.

***Mechanism of action.*** Brain imaging studies have demonstrated metabolic changes in the prefrontal cortex and in limbic structures relevant to mood regulation ^62^, possibly through the modulation of monoaminergic neurotransmission ^63^.

***Clinical application.*** VNS, in its current form, is a chronic treatment. During the first months of treatment, the best stimulation parameters have to be selected; therefore, regular visits are required at the beginning of therapy. In the long-term, yearly checkups are advised to ensure the functioning of the device (e.g., battery exhaustion and lead connection) and to adjust parameters if necessary.

***Efficacy.*** In 2005, the FDA-approved VNS therapy for the adjunctive long-term treatment of chronic or recurrent depression for those patients who have not had an adequate response to two or more antidepressant treatments.

Long-term effects were significantly superior by outcomes in comparison to patients receiving treatment as usual. However, VNS therapy is more effective in patients with moderate but not extreme levels of resistance ^64, 65^ (see Table 1).

***Side effects.*** Possible side-effects of VNS therapy are: an infection at the device, a hoarse voice, cough, and shortness of breath, as well as difficulties in swallowing ^64, 65^.

***Ethical aspects.*** Although clinical efficacy has been proven and is superior to non-invasive treatments (e.g., repetitive TMS), VNS is not available for many patients as insurance companies only cover the cost for the surgery and not for the (psychiatric and neurosurgical) long-term treatment. For financial reasons, VNS is therefore unattractive to hospitals. This situation contravenes the criteria of justice, well-being and autonomy. In addition, the opportunity to conduct research is limited and important safety aspects (e.g., predictors of response and long-term side-effects) are not assessed sufficiently. This again contravenes the criterion of patient well-being.

***Current research and outlook.*** Research in VNS is limited because of the above mentioned financial restraints. Predictors of response (prior response to ECT, age, subtypes of depression etc.) and long-term safety (above 3 years) can only be inferred from its use in the treatment of epilepsy.

### Deep brain stimulation

***Method.*** DBS is the most invasive neuromodulation technique because it involves the stereotactic implantation of unilateral or bilateral electrodes in the brain, connected to a permanently implanted, battery-powered neurostimulator. Usually, a pair of electrodes are placed into a specific brain region assumed to be involved in mood regulation. Constant stimulation can be adjusted with the parameters of voltage, pulse width, frequency and shape of the electric field.

***Mechanism of action.*** The effect of DBS on the brain is far from being understood. Stimulation parameters (frequency, amplitude, pulse width, duration) also clearly have an impact on the effect. With commonly used parameters, a relatively large volume of neural tissue is influenced ^66^.

Functional neuroimaging data have demonstrated that DBS changes the activity of brain areas far beyond the targeted region. Thus complex neural networks are putatively modulated ^66– 68^.

In hypothesis-guided approaches, several brain structures are targets of DBS: the subgenual cingulate gyrus (Cg25) ^32, 67, 69^, the anterior limb of the capsula interna (ALIC) ^70, 71^ and the nucleus accumbens (Nacc) ^68, 72^, and the supero-lateral branch of the medial forebrain bundle (slMFB) ^73^.

***Clinical application.*** DBS is only available for a highly selected group of patients suffering from very therapy-resistant depression in clinical studies in a few centers worldwide. Launching a DBS study requires a specialized multidisciplinary team, including a psychiatrist, psychologist, and neurosurgeon, and the possibility for a long-term follow-up.

***Efficacy.*** In small pilot studies, an antidepressant effect of DBS was described: a reduction of symptoms of greater than 50% was reached in about 50% of the patients after 12 months of DBS treatment ^70, 74– 78^. First results have found superior response rates in the slMFB (more rapid effects and >70% response rates after 3 months ^79^ and after 12 months ^79^), but long-term data and larger samples are required for efficacy evaluation. First small studies with sham stimulation found conflicting results concerning placebo effects ^79, 80^ (see Table 1).

***Side effects.*** The adverse reactions caused by DBS can be differentiated in first effects related to the surgical implantation procedure itself (e.g., bleeding, infection) and second effects related to the stimulation which depend on the target site of stimulation (e.g., paresthesia, muscle contraction, dysarthria, and diplopia, hypomania, anxiety). The former are rare (i.e., risk of seizure 1–3%, of bleeding 1–5%, and of infection 2–25%), the latter are reversible with a parameter adjustment. DBS seems to have neutral-to-positive effects regarding cognition ^33, 74, 75^.

***Ethical aspects.*** Few treatment approaches in psychiatry have initiated as much ethical debate as DBS. Major issues concerning patient autonomy are: the manipulation of human personality with DBS ^81^, a sudden disruption of the patient’s biography ^82^, and the ability of patients with treatment-resistant depression to give informed consent ^83^.

Regarding well-being, the induction of new psychiatric symptoms (e.g., hypomania symptoms ^84^ or high-risk behavior ^85^) is debatable. Because DBS is a high-risk intervention, patients have to be carefully selected and, as long as the optimal target has not been established and efficacy is questionable, only patients resistant to all conventional treatment approaches (including ECT) should be selected for studies. Careful individual risk-benefit ratios are necessary to ensure the criterion of patient well-being.

The idea of possibly enhancing cognitive functions is important in terms of the criterion of justice, although in treatment-resistant depression, the amelioration of cognitive functions could be discussed in relation to a prior dysfunction in cognition caused by the depression ^81^.

DBS is only available in specialized centers for a few, highly selected, therapy-resistant patients. In addition, DBS is very expensive and dominated by a few companies, and little investment from the government exists. These factors restrict availability but should be seen in the light of patient well-being (e.g., to prevent harm from untrained staff, and random application before safety is assessed).

***Current research and outlook.*** DBS is a treatment method in the early phase of evaluation (level III). Therefore, efficacy and safety have to be assessed in sham stimulation control designs. Due to ethical reasons, it is difficult to install a randomized design. Current research questions are the best target site, parameter adjustment protocols, the predictive value of acute stimulation effects, and other predictors of response (e.g., depression subclusters, length and number of depressive episodes, former response to ECT). Furthermore, imaging studies are necessary to elucidate the mode of action.

## Summary and outlook

In the last two decades, many neuromodulation techniques have evolved at different levels of evidence. Noninvasive techniques (ECT, MST and TMS) and invasive techniques (VNS and DBS) with different safety profiles, as well as limited data on long-term efficacy and reduced availability, make it a challenge to select an appropriate treatment for patients with treatment-resistant depression.

Illness chronicity, severity of the current episode, as well as nonresponse to other treatment approaches and fear of side effects, should be considered among other factors. After all, it is also the patient’s choice if an evaluated treatment with a very good short-term efficacy but inferior side-effect profile (e.g., ECT) is preferred, rather than a more experimental treatment with a possibly favorable side-effect profile (e.g., MST), or an experimental treatment with lower response rates (e.g., TMS). For more resistant courses of treatment-resistant depression (e.g., after non-response to TMS and ECT), VNS can be an option. At the current stage of research, DBS should only be offered to extremely treatment-resistant patients with limited psychiatric comorbidity within clinical studies in order to protect patient well-being.

If available, the treatment associated with the best side-effect profile and efficacy should be selected. Reality shows that, due to reduced availability and lacking long-term efficacy data, most patients with treatment-resistant depression face a trial-and-error approach to therapeutics. This contravenes the ethical criteria of patient autonomy and justice. There is minimal guidance for clinicians concerning long-term management of these complex patients. This is inefficient, costly, and associated with poor outcomes, and patients are facing a reduced quality of life. It is therefore necessary to support long-term research in neuromodulation for treatment-resistant depression and to conduct large sample randomized control trials. This only seems possible with public funding in addition to company-sponsored trials. This would allow us to raise the level of evidence for neuromodulation treatments as promising therapy options for treatment-resistant depression.

## Abbreviations

DBS, deep brain stimulation; ECT, electroconvulsive therapy; MST, magnetic seizure therapy; slMFB, supero-lateral branch of the medial forebrain bundle; TMS, transcranial magnetic stimulation; VNS, vagus nerve stimulation.

## References

[1] Rush AJ Trivedi MH Wisniewski SR: Acute and longer-term outcomes in depressed outpatients requiring one or several treatment steps: a STAR*D report. *Am J Psychiatry.* 2006;163(11):1905–17. 10.1176/appi.ajp.163.11.1905 17074942

[2] Rush AJ Warden D Wisniewski SR: STAR*D: revising conventional wisdom. *CNS Drugs.* 2009;23(8):627–47. 10.2165/00023210-200923080-00001 19594193

[3] Sackeim HA: The definition and meaning of treatment-resistant depression. *J Clin Psychiatry.* 2001;62(Suppl 16):10–7. 11480879

[4] Thase ME Rush AJ: When at first you don't succeed: sequential strategies for antidepressant nonresponders. *J Clin Psychiatry.* 1997;58(Suppl 13):23–9. 9402916

[5] Schlaepfer TE Agren H Monteleone P: The hidden third: improving outcome in treatment-resistant depression. *J Psychopharmacol.* 2012;26(5):587–602. 10.1177/0269881111431748 22236505

[6] Folstein MF Folstein SE McHugh PR: "Mini-mental state". A practical method for grading the cognitive state of patients for the clinician. *J Psychiatric Res.* 1975;12(3):189–98. 10.1016/0022-3956(75)90026-6 1202204

[7] Nierenberg AA: Long-term management of chronic depression. *J Clin Psychiatry.* 2001;62(Suppl 6):17–21. 11310815

[8] Fekadu A Wooderson SC Markopoulo K: What happens to patients with treatment-resistant depression? A systematic review of medium to long term outcome studies. *J Affect Disord.* 2009;116(1–2):4–11. 10.1016/j.jad.2008.10.014 19007996

[9] Warden D Rush AJ Wisniewski SR: What predicts attrition in second step medication treatments for depression?: a STAR*D Report. *Int J Neuropsychopharmacol.* 2009;12(4):459–73. 10.1017/S1461145708009073 18611293 PMC5885751

[10] Kennedy SH Lam RW Parikh SV: Canadian Network for Mood and Anxiety Treatments (CANMAT) clinical guidelines for the management of major depressive disorder in adults. Introduction. *J Affect Disord.* 2009;117(Suppl 1):S1–2. 10.1016/j.jad.2009.06.043 19682750

[11] Illes J Bird SJ: Neuroethics: a modern context for ethics in neuroscience. *Trends Neurosci.* 2006;29(9):511–7. 10.1016/j.tins.2006.07.002 16859760 PMC1656950

[12] Weiner RD Krystal AD: EEG monitoring and management of electrically induzed seizures. Washington, DC, American Psychiatric Press,1993. 1771151

[13] Krystal AD Weiner RD Coffey CE: The ictal EEG as a marker of adequate stimulus intensity with unilateral ECT. *J Neuropsychiatry Clin Neurosci.* 1995;7(3):295–303. 10.1176/jnp.7.3.295 7580187

[14] Sackeim HA: The anticonvulsant hypothesis of the mechanisms of action of ECT: current status. *J ECT.* 1999;15(1):5–26. 10.1097/00124509-199903000-00003 10189616

[15] Krystal AD West M Prado R: EEG effects of ECT: implications for rTMS. *Depress Anxiety.* 2000;12(3):157–65. 10.1002/1520-6394(2000)12:3<157::AID-DA7>3.0.CO;2-R 11126190

[16] Luber B Nobler MS Moeller JR: Quantitative EEG during seizures induced by electroconvulsive therapy: relations to treatment modality and clinical features. II. Topographic analyses. *J ECT.* 2000;16(3):229–43. 10.1097/00124509-200009000-00003 11005044

[17] Nobler MS Luber B Moeller JR: Quantitative EEG during seizures induced by electroconvulsive therapy: relations to treatment modality and clinical features. I. Global analyses. *J ECT.* 2000;16(3):211–28. 10.1097/00124509-200009000-00002 11005043

[18] Sackeim HA Haskett RF Mulsant BH: Continuation pharmacotherapy in the prevention of relapse following electroconvulsive therapy: a randomized controlled trial. *JAMA.* 2001;285(10):1299–307. 10.1001/jama.285.10.1299 11255384

[19] Khalid N Atkins M Tredget J: The effectiveness of electroconvulsive therapy in treatment-resistant depression: a naturalistic study. *J ECT.* 2008;24(2):141–5. 10.1097/YCT.0b013e318157ac58 18580559

[20] APA, Ed: Diagnostic and Statistical Manual of Mental Disorders (DSM IV). Washington DC, American Psychiatric Association,1994 Reference Source

[21] Ebmeier KP Donaghey C Steele JD: Recent developments and current controversies in depression. *Lancet.* 2006;367(9505):153–67. 10.1016/S0140-6736(06)67964-6 16413879

[22] Lipsman N Sankar T Downar J: Neuromodulation for treatment-refractory major depressive disorder. *CMAJ.* 2014;186(1):33–9. 10.1503/cmaj.121317 23897945 PMC3883821

[23] Donahue AB: Electroconvulsive therapy and memory loss: a personal journey. *J ECT.* 2000;16(2):133–43. 10868323 10.1097/00124509-200006000-00005

[24] Datka W Siwek M Dudek D: [Working memory disturbances in patients with major depression after ECT treatment]. *Psychiatr Pol.* 2007;41(3):339–49. 17900050

[25] Rose D Fleischmann P Wykes T: Patients' perspectives on electroconvulsive therapy: systematic review. *BMJ.* 2003;326(7403):1363. 10.1136/bmj.326.7403.1363 12816822 PMC162130

[26] Rapoport MJ Mamdani M Herrmann N: Electroconvulsive therapy in older adults: 13-year trends. *Can J Psychiatry.* 2006;51(9):616–9. 17007229 10.1177/070674370605100910

[27] Payne NA Prudic J: Electroconvulsive therapy: Part I. A perspective on the evolution and current practice of ECT. *J Psychiatr Pract.* 2009;15(5):346–68. 10.1097/01.pra.0000361277.65468.ef 19820553 PMC3042260

[28] Payne NA Prudic J: Electroconvulsive therapy: Part II: a biopsychosocial perspective. *J Psychiatr Pract.* 2009;15(5):369–90. 10.1097/01.pra.0000361278.73092.85 19820554 PMC3069072

[29] Kayser S Bewernick BH Hurlemann R: Comparable seizure characteristics in magnetic seizure therapy and electroconvulsive therapy for major depression. *Eur Neuropsychopharmacol.* 2013;23(11):1541–50. 10.1016/j.euroneuro.2013.04.011 23820052

[30] Soehle M Kayser S Ellerkmann RK: Bilateral bispectral index monitoring during and after electroconvulsive therapy compared with magnetic seizure therapy for treatment-resistant depression. *Br J Anaesth.* 2014;112(4):695–702. 10.1093/bja/aet410 24305645

[31] Austin MP Mitchell P Goodwin GM: Cognitive deficits in depression: possible implications for functional neuropathology. *Br J Psychiatry.* 2001;178(3):200–6. 10.1192/bjp.178.3.200 11230029

[32] McNeely HE Mayberg HS Lozano AM: Neuropsychological impact of Cg25 deep brain stimulation for treatment-resistant depression: preliminary results over 12 months. *J Nerv Ment Dis.* 2008;196(5):405–10. 10.1097/NMD.0b013e3181710927 18477883

[33] Grubert C Hurlemann R Bewernick BH: Neuropsychological safety of nucleus accumbens deep brain stimulation for major depression: effects of 12-month stimulation. *World J Biol Psychiatry.* 2011;12(7):516–27. 10.3109/15622975.2011.583940 21736514

[34] Lisanby SH Schlaepfer TE Fisch HU: Magnetic seizure therapy of major depression. *Arch Gen Psychiatry.* 2001;58(3):303–5. 10.1001/archpsyc.58.3.303 11231838

[35] Rowny SB Cycowicz YM McClintock SM: Differential heart rate response to magnetic seizure therapy (MST) relative to electroconvulsive therapy: a nonhuman primate model. *Neuroimage.* 2009;47(3):1086–91. 10.1016/j.neuroimage.2009.05.070 19497373 PMC3674813

[36] Kosel M Frick C Lisanby SH: Magnetic seizure therapy improves mood in refractory major depression. *Neuropsychopharmacology.* 2003;28(11):2045–8. 10.1038/sj.npp.1300293 12942146

[37] Hoy KE Thomson RH Cherk M: Effect of magnetic seizure therapy on regional brain glucose metabolism in major depression. *Psychiatry Res.* 2013;211(2):169–75. 10.1016/j.pscychresns.2012.08.003 23149039

[38] Kayser S Bewernick BH Matusch A: Magnetic seizure therapy in treatment-resistant depression: clinical, neuropsychological and metabolic effects. *Psychol Med.* 2015;45(5):1073–92. 10.1017/S0033291714002244 25420474

[39] Lisanby SH Luber B Schlaepfer TE: Safety and feasibility of magnetic seizure therapy (MST) in major depression: randomized within-subject comparison with electroconvulsive therapy. *Neuropsychopharmacology.* 2003;28(10):1852–65. 10.1038/sj.npp.1300229 12865903

[40] White PF Amos Q Zhang Y: Anesthetic considerations for magnetic seizure therapy: a novel therapy for severe depression. *Anesth Analg.* 2006;103(1):76–80, table of contents. 10.1213/01.ane.0000221182.71648.a3 16790630

[41] Kayser S Bewernick B Axmacher N: Magnetic seizure therapy of treatment-resistant depression in a patient with bipolar disorder. *J ECT.* 2009;25(2):137–40. 10.1097/YCT.0b013e31817dc45a 19057399

[42] Kayser S Bewernick BH Grubert C: Antidepressant effects, of magnetic seizure therapy and electroconvulsive therapy, in treatment-resistant depression. *J Psychiatr Res.* 2011;45(5):569–76. 10.1016/j.jpsychires.2010.09.008 20951997

[43] Fitzgerald PB Hoy KE Herring SE: Pilot study of the clinical and cognitive effects of high-frequency magnetic seizure therapy in major depressive disorder. *Depress Anxiety.* 2013;30(2):129–36. 10.1002/da.22005 23080404

[44] Bersani FS Girardi N Sanna L: Deep transcranial magnetic stimulation for treatment-resistant bipolar depression: a case report of acute and maintenance efficacy. *Neurocase.* 2013;19(5):451–7. 10.1080/13554794.2012.690429 22827578

[45] Roth Y Zangen A Hallett M: A coil design for transcranial magnetic stimulation of deep brain regions. *J Clin Neurophysiol.* 2002;19(4):361–70. 10.1097/00004691-200208000-00008 12436090

[46] Roth Y Amir A Levkovitz Y: Three-dimensional distribution of the electric field induced in the brain by transcranial magnetic stimulation using figure-8 and deep H-coils. *J Clin Neurophysiol.* 2007;24(1):31–8. 10.1097/WNP.0b013e31802fa393 17277575

[47] George MS Nahas Z Lisanby SH: Transcranial magnetic stimulation. *Neurosurg Clin N Am.* 2003;14(2):283–301. 10.1016/S1042-3680(02)00120-1 12856495

[48] Fitzgerald PB Fountain S Daskalakis ZJ: A comprehensive review of the effects of rTMS on motor cortical excitability and inhibition. *Clin Neurophysiol.* 2006;117(12):2584–96. 10.1016/j.clinph.2006.06.712 16890483

[49] Levkovitz Y Harel EV Roth Y: Deep transcranial magnetic stimulation over the prefrontal cortex: evaluation of antidepressant and cognitive effects in depressive patients. *Brain Stimul.* 2009;2(4):188–200. 10.1016/j.brs.2009.08.002 20633419

[50] Levkovitz Y Sheer A Harel EV: Differential effects of deep TMS of the prefrontal cortex on apathy and depression. *Brain Stimul.* 2011;4(4):266–74. 10.1016/j.brs.2010.12.004 22032742

[51] Harel EV Rabany L Deutsch L: H-coil repetitive transcranial magnetic stimulation for treatment resistant major depressive disorder: An 18-week continuation safety and feasibility study. *World J Biol Psychiatry.* 2014;15(4):298–306. 10.3109/15622975.2011.639802 22313023

[52] Gaynes BN Lloyd SW Lux L: Repetitive transcranial magnetic stimulation for treatment-resistant depression: a systematic review and meta-analysis. *J Clin Psychiatry.* 2014;75(5):477–89; quiz 489. 10.4088/JCP.13r08815 24922485

[53] Levkovitz Y Roth Y Harel EV: A randomized controlled feasibility and safety study of deep transcranial magnetic stimulation. *Clin Neurophysiol.* 2007;118(12):2730–44. 10.1016/j.clinph.2007.09.061 17977787

[54] Rosenberg O Zangen A Stryjer R: Response to deep TMS in depressive patients with previous electroconvulsive treatment. *Brain Stimul.* 2010;3(4):211–7. 10.1016/j.brs.2009.12.001 20965450

[55] Harel EV Zangen A Roth Y: H-coil repetitive transcranial magnetic stimulation for the treatment of bipolar depression: an add-on, safety and feasibility study. *World J Biol Psychiatry.* 2011;12(2):119–26. 10.3109/15622975.2010.510893 20854181

[56] Isserles M Rosenberg O Dannon P: Cognitive-emotional reactivation during deep transcranial magnetic stimulation over the prefrontal cortex of depressive patients affects antidepressant outcome. *J Affect Disord.* 2011;128(3):235–42. 10.1016/j.jad.2010.06.038 20663568

[57] Rosenberg O Isserles M Levkovitz Y: Effectiveness of a second deep TMS in depression: a brief report. *Prog Neuropsychopharmacol Biol Psychiatry.* 2011;35(4):1041–4. 10.1016/j.pnpbp.2011.02.015 21354242

[58] Little JT Kimbrell TA Wassermann EM: Cognitive effects of 1- and 20-hertz repetitive transcranial magnetic stimulation in depression: preliminary report. *Neuropsychiatry Neuropsychol Behav Neurol.* 2000;13(2):119–24. 10780630

[59] Schulze-Rauschenbach SC Harms U Schlaepfer TE: Distinctive neurocognitive effects of repetitive transcranial magnetic stimulation and electroconvulsive therapy in major depression. *Br J Psychiatry.* 2005;186:410–6. 10.1192/bjp.186.5.410 15863746

[60] Janicak PG O'Reardon JP Sampson SM: Transcranial magnetic stimulation in the treatment of major depressive disorder: a comprehensive summary of safety experience from acute exposure, extended exposure, and during reintroduction treatment. *J Clin Psychiatry.* 2008;69(2):222–32. 10.4088/JCP.v69n0208 18232722

[61] Schlaepfer TE George MS Mayberg H: WFSBP Guidelines on Brain Stimulation Treatments in Psychiatry. *World J Biol Psychiatry.* 2010;11(1):2–18. 10.3109/15622970903170835 20146648

[62] Drevets WC Bogers W Raichle ME: Functional anatomical correlates of antidepressant drug treatment assessed using PET measures of regional glucose metabolism. *Eur Neuropsychopharmacol.* 2002;12(6):527–44. 10.1016/S0924-977X(02)00102-5 12468016

[63] Dorr AE Debonnel G: Effect of vagus nerve stimulation on serotonergic and noradrenergic transmission. *J Pharmacol Exp Ther.* 2006;318(2):890–8. 10.1124/jpet.106.104166 16690723

[64] Nahas Z Marangell LB Husain MM: Two-year outcome of vagus nerve stimulation (VNS) for treatment of major depressive episodes. *J Clin Psychiatry.* 2005;66(9):1097–104. 10.4088/JCP.v66n0902 16187765

[65] Schlaepfer TE Frick C Zobel A: Vagus nerve stimulation for depression: efficacy and safety in a European study. *Psychol Med.* 2008;38(5):651–61. 10.1017/S0033291707001924 18177525

[66] Kringelbach ML Jenkinson N Owen SL: Translational principles of deep brain stimulation. *Nat Rev Neurosci.* 2007;8(8):623–35. 10.1038/nrn2196 17637800

[67] Mayberg HS Lozano AM Voon V: Deep brain stimulation for treatment-resistant depression. *Neuron.* 2005;45(5):651–60. 10.1016/j.neuron.2005.02.014 15748841

[68] Bewernick BH Hurlemann R Matusch A: Nucleus accumbens deep brain stimulation decreases ratings of depression and anxiety in treatment-resistant depression. *Biol Psychiatry.* 2010;67(2):110–6. 10.1016/j.biopsych.2009.09.013 19914605

[69] Lozano AM Mayberg HS Giacobbe P: Subcallosal cingulate gyrus deep brain stimulation for treatment-resistant depression. *Biol Psychiatry.* 2008;64(6):461–7. 10.1016/j.biopsych.2008.05.034 18639234

[70] Malone DA Jr Dougherty DD Rezai AR: Deep brain stimulation of the ventral capsule/ventral striatum for treatment-resistant depression. *Biol Psychiatry.* 2009;65(4):267–75. 10.1016/j.biopsych.2008.08.029 18842257 PMC3486635

[71] Malone DA Jr: Use of deep brain stimulation in treatment-resistant depression. *Cleve Clin J Med.* 2010;77(Suppl 3):S77–80. 10.3949/ccjm.77.s3.14 20622083

[72] Schlaepfer TE Cohen MX Frick C: Deep brain stimulation to reward circuitry alleviates anhedonia in refractory major depression. *Neuropsychopharmacology.* 2008;33(2):368–77. 10.1038/sj.npp.1301408 17429407

[73] Schlaepfer TE Bewernick BH Kayser S: Deep brain stimulation of the human reward system for major depression--rationale, outcomes and outlook. *Neuropsychopharmacology.* 2014;39(6):1303–14. 10.1038/npp.2014.28 24513970 PMC3988559

[74] Kennedy SH Giacobbe P Rizvi SJ: Deep brain stimulation for treatment-resistant depression: follow-up after 3 to 6 years. *Am J Psychiatry.* 2011;168(5):502–10. 10.1176/appi.ajp.2010.10081187 21285143

[75] Bewernick BH Kayser S Sturm V: Long-term effects of nucleus accumbens deep brain stimulation in treatment-resistant depression: evidence for sustained efficacy. *Neuropsychopharmacology.* 2012;37(9):1975–85. 10.1038/npp.2012.44 22473055 PMC3398749

[76] Holtzheimer PE Kelley ME Gross RE: Subcallosal cingulate deep brain stimulation for treatment-resistant unipolar and bipolar depression. *Arch Gen Psychiatry.* 2012;69(2):150–8. 10.1001/archgenpsychiatry.2011.1456 22213770 PMC4423545

[77] Lozano AM Giacobbe P Hamani C: A multicenter pilot study of subcallosal cingulate area deep brain stimulation for treatment-resistant depression. *J Neurosurg.* 2012;116(2):315–22. 10.3171/2011.10.JNS102122 22098195

[78] Puigdemont D Pérez-Egea R Portella MJ: Deep brain stimulation of the subcallosal cingulate gyrus: further evidence in treatment-resistant major depression. *Int J Neuropsychopharmacol.* 2012;15(1):121–33. 10.1017/S1461145711001088 21777510

[79] Schlaepfer TE: Rewiring faulty circuits-the promise of deep brain stimulation for psychiatry. Euro Global Summit and Medicare Expo on Psychiatry. Barcelona, Spain, OMICS International Conference,2015 10.4172/2378-5756.S1.001

[80] Dougherty D Carpenter L Bhati M: 720-A Randomized Sham-Controlled Trial of DBS of the VC/VS for Treatment-Resistant Depression. *Biol Psychiatry.* 2012;71(8):230S. 10.1016/j.biopsych.2014.11.023 25726497

[81] Synofzik M Schlaepfer TE: Stimulating personality: ethical criteria for deep brain stimulation in psychiatric patients and for enhancement purposes. *Biotechnol J.* 2008;3(12):1511–20. 10.1002/biot.200800187 19072907

[82] Gisquet E: Cerebral implants and Parkinson's disease: a unique form of biographical disruption? *Soc Sci Med.* 2008;67(11):1847–51. 10.1016/j.socscimed.2008.09.026 18947914

[83] Appelbaum PS Grisso T Frank E: Competence of depressed patients for consent to research. *Am J Psychiatry.* 1999;156(9):1380–4. 10484948 10.1176/ajp.156.9.1380

[84] Synofzik M Schlaepfer TE Fins JJ: How Happy Is Too Happy? Euphoria, Neuroethics, and Deep Brain Stimulation of the Nucleus Accumbens. *AJOB Neurosci.* 2012;3(1):30–6. 10.1080/21507740.2011.635633

[85] Frank MJ Samanta J Moustafa AA: Hold your horses: impulsivity, deep brain stimulation, and medication in parkinsonism. *Science.* 2007;318(5854):1309–12. 10.1126/science.1146157 17962524

